# State-of-the-Art and Opportunities for Bioactive Pentacyclic Triterpenes from Native Mexican Plants

**DOI:** 10.3390/plants11172184

**Published:** 2022-08-23

**Authors:** Juan Antonio Alfaro-Almaguer, Luis Alberto Mejía-Manzano, José González-Valdez

**Affiliations:** School of Engineering and Science, Tecnologico de Monterrey, Campus Monterrey, Monterrey 64849, Mexico

**Keywords:** pentacyclic triterpenes, Mexican plants, biological activity, extraction, isolation, drug delivery

## Abstract

Native Mexican plants are a wide source of bioactive compounds such as pentacyclic triterpenes. Pentacyclic triterpenes biosynthesized through the mevalonate (MVA) and the 2-C-methyl-D-erythritol-phosphate (MEP) metabolic pathways are highlighted by their diverse biological activity. Compounds belonging to the oleanane, ursane, and lupane groups have been identified in about 33 Mexican plants, located geographically in the southwest of Mexico. The works addressing these findings have reported 45 compounds that mainly show antimicrobial activity, followed by anti-inflammatory, cytotoxic, anxiolytic, hypoglycemic, and growth-stimulating or allelopathic activities. Extraction by maceration and Soxhlet with organic solvents and consecutive chromatography of silica gel have been used for their whole or partial purification. Nanoparticles and nanoemulsions are the vehicles used in Mexican formulations for drug delivery of the pentacyclic triterpenes until now. Sustainable extraction, formulation, regulation, isolation, characterization, and bioassay facilities are areas of opportunity in pentacyclic triterpenes research in Mexico while the presence of plant and human resources and traditional knowledge are strengths. The present review discusses the generalities of the pentacyclic triterpene (definition, biogenic classification, and biosynthesis), a summary of the last two decades of research on the compounds identified and their evaluated bioactivity, the generalities about the extraction and purification methods used, drug delivery aspects, and a critical analysis of the advantages and limitations of research carried out in this way.

## 1. Introduction

Mexico belongs to the “megadiverse” group of countries and due in part to its wide plant biodiversity. It is estimated that Mexico possesses between 21,989 and 23,424 vascular plant species, placing the country in the 5th position within this category [1,2]. Approximately, 40% of this flora is endemic and 17% (around 4000 species) is believed to have medicinal properties [3]. Generally, medicinal plants have a wild origin and many of these have been employed for centuries for the treatment of diverse illnesses ranging from acute problems to severe or chronic disorders. However, not all these plants have been discovered, characterized, and/or studied.

It is well known that medicinal plants are an inexhaustible source of chemical compounds with potential therapeutic effects. In this context, one of the groups of phytochemicals highlighted by their diverse biological activity are triterpenes, which have displayed anti-inflammatory, analgesic, antibiotic, antiviral, antimycotic, immunomodulator, hepatoprotective, hemolytic, cytostatic, and anticancer properties, the latter by exerting antiangiogenic, proapoptotic, and prodifferentiative effects [4,5]. Specifically, pentacyclic triterpenes (PCTs) are a subgroup of triterpenes that present some of these properties and are attracting scientific attention for their potential use as therapeutic agents [6,7]. For these reasons, this review aims to offer an updated analysis about PCT research on Mexican plants during the last 30 years, covering aspects such as the tested biological activity, used isolation and purification techniques, and formulation alternatives. In the first section, the chemical concept, classification, and generalities of PCTs are shown.

### 1.1. General Overview of Pentacyclic Triterpenes

#### 1.1.1. Structure, Classification, Distribution, and Biological Importance

Pentacyclic triterpenes (PCTs) are subcategorized inside the triterpenoid cluster and are heterogeneous compounds of 30 carbon atoms [8] belonging to the general group of compounds called terpenes, whose basic subunit is isoprene (methyl-1,3-butadiene). In general, terpenes composed of two isoprene units are called monoterpenoids (10 C), those with three units are known as sesquiterpenoids (15 C), those with four units as diterpenes (20 C), and those with more than four subunits are classified as polyterpenes [9]. Triterpenes are considered polyterpenes because they present six isoprene units. Both sesquiterpenes and polyterpenes are produced in the plant’s cytosol while the majority of diterpenes and tetraterpenes (40C) have their origin in plastids [10].

**Figure 1:**
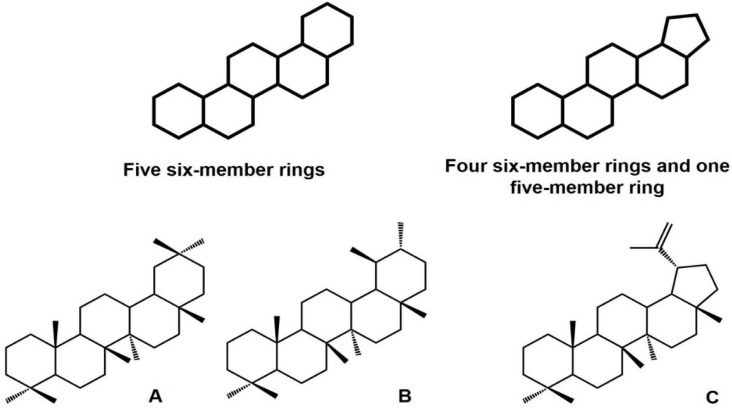
Pentacyclic triterpene backbone classification and representative subgroups: oleanane (**A**), ursane (**B**), and lupane (**C**).

On their part, PCTs are molecules with a backbone based on five six-member rings or four cyclohexanes and one cyclopentane. Ursane, oleanane, taraxastane, gammacerane, and friedelane are PCTs made from five six-member rings while lupane and hopane are groups based on four six-member rings and a final five-member ring (Figure 1) [6,11,12]. The classification criteria for the establishment of these triterpene groups are based on biogenetic principles [11]. PCTs are widely distributed in nature and are present in fruit peel, stem bark, and leaves [11,13]. In addition, these can be found in their free form, as esters or as saponins (i.e., combined with other sugars) [11].

In the last years, several beneficial activities of PCTs with medical importance have been described, including anti-inflammatory, antibacterial, antiviral, anti-parasitic, anticancerogenic, antidepressant, anti-anxiety, neuroprotective, cardioprotective, and immunomodulatory effects [6,14,15,16] Moreover, triterpenes from the lupane, oleanane, and ursane groups have received special attention because of their pharmacological effects, coupled with their low toxicity [13]. Naturally, PCTs are produced as a defense against metabolic oxidations and some other factors such as microorganisms or environmental stress [17]. The bioactivity of the triterpenes is highly affected by its backbone and the position and number of chemical groups (i.e., alkyl, hydroxyl, carbonyl, and aminoacids) in their structure [18]. It is important to point out that many of these PCTs, because of the complexity of their structure, have not yet been chemically synthesized.

#### 1.1.2. Biosynthesis

**Figure 2:**
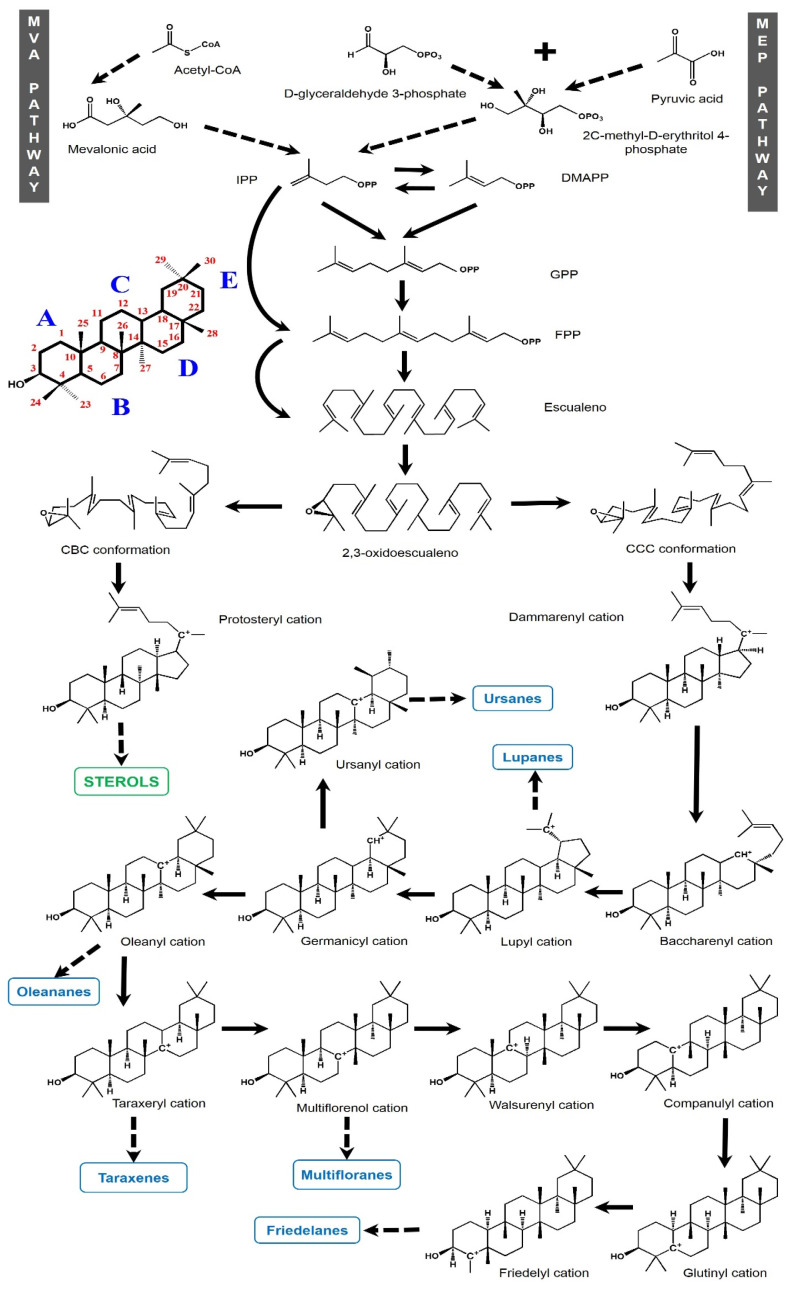
Biosynthetic pathway of PCTs in plants through the mevalonate (MVA) pathway and 2-C-methyl-D-erythritol-phosphate (MEP) pathway, showing the initial substrates, squalene synthesis, and cyclizing and rearrangement of cations that result in the diverse groups of PCTs.

The isoprene units integrating triterpenes are biosynthesized by two metabolic pathways: the mevalonate (MVA) pathway and the 2-C-methyl-D-erythritol-phosphate (MEP) pathway. The MVA pathway occurs in the cytosol from pyruvate and glyceraldehyde 3-phosphate while the MEP pathway occurs in the plastids [19] from 3 acetyl-CoA molecules [20]. The objective of both routes is to produce IPP (isopentenyl diphosphate), a molecule that contains the required isoprene backbone for terpenoid synthesis (Figure 2). On its part, this backbone can be transported between plastids and the cytosol, enabling interactions between both routes [21]. IPP is isomerized to dimethylallyl diphosphate (DMAPP), and this isomer is condensed with another molecule of IPP to produce geranyl diphosphate (GPP), a compound of 10 carbons. If another IPP molecule is added to GPP, farnesyl diphosphate (FPP) with 15 carbons is obtained. The subsequent addition of an IPP unit generates the 20-carbon geranylgeranyl diphosphate (GGPP). These building blocks are the precursor structures of the different terpene groups [22]. The combination of two FPP units through the squalene synthase enzyme forms squalene (30 carbons). In the next step, squalene is turned into squalene epoxide (2,3-oxidosqualene) by squalene monoxigenase and the latter is cyclized with a subsequent structure rearrangement [11,23], which can be turned into a variety of polycyclic triterpenes depending on the enzymes involved, called oxidosqualene cyclases (OSCs) [24]. However, prior to the action of OSCs, the 2,3-oxidosqualene must adopt a chair-chair-chair (CCC) conformation, with this being a key step in which the synthesis is oriented towards triterpenes, having the dammarenyl cation as an initial intermediate. Otherwise, if the 2,3-oxidosqualene adopts a chair-boat-chair (CBC) conformation, the synthesis is directed to sterols through the protosteryl cation as the main intermediary [23]. Recently, this dichotomy has been questioned because a mutant rice OSC (which normally produces a sterol) yielded a novel PCT with an architecture that is more related to the dammarenyl cation or even to an undescribed third cation [25].

Considering the formation of PTCs from the CCC conformation, OSCs start the cyclization with the protonation of the final double bond in the 2,3-oxidosqualene. The variation in carbocation cyclization and backbone rearrangements generate the diverse groups of PTCs [23,26]. So, the dammarenyl cation can be expanded from C20 to C18, forming a baccharenyl cation. Another ring expansion to C20 generates a compound of five rings known as the lupyl cation [27], which is the backbone of the lupane group. When the E ring in the lupyl cation suffers an expansion and the carbocation migrates to C19, the germanicyl cation is formed. From this intermediary, the cation re-location to C14 and a methyl shift produces the precursor of ursanes. Additionally, the oleanyl cation is rendered from the germanicyl cation, containing the C13 cation in the same way as the ursanyl cation but with two methyl groups in the C20 position instead of a methyl group in C19 and another in C20. The next biosynthesized backbone intermediary from the oleanyl cation is the taraxeryl cation with the cation in C14. If the carbocation migrates to C8, the multiflorenyl cation is produced. When the carbocation is in C9 or C10, the walsurenyl or companulyl cations are produced, respectively. The displacement to the C5 results in a glutinyl cation. Finally, the last PCT and most rearranged (friedelanes) group is formed through the friedelyl cation with the carbocation in the C4 position (Figure 2).

OSCs are encoded by many genes in plants and many of these enzymes may participate in the formation of multiple products [23]. The structure diversification in these groups of PCTs is achieved by modifications of each scaffold base such as oxygenation (i.e., addition of functional groups carbonyl, aldehyl, hydroxyl, carboxyl, and epoxy groups), glycosylation, and acylation. The oxygenation reactions are catalyzed by cytochrome P450 monooxygenases (P450s). Until 2017, about 55 P450s related to PCT biosynthesis in plants had been identified, which belonged to the CYP716, CYP51, CYP71, CYP72, CYP87, CYP88, and CYP93 families [28]. It is important to clarify that complete biosynthetic pathways of specific PCTs have not been completely elucidated yet; hence, a large amount of PCTs may still be discovered.

## 2. Pentacyclic Triterpenes from Mexican Plants

**Figure 3:**
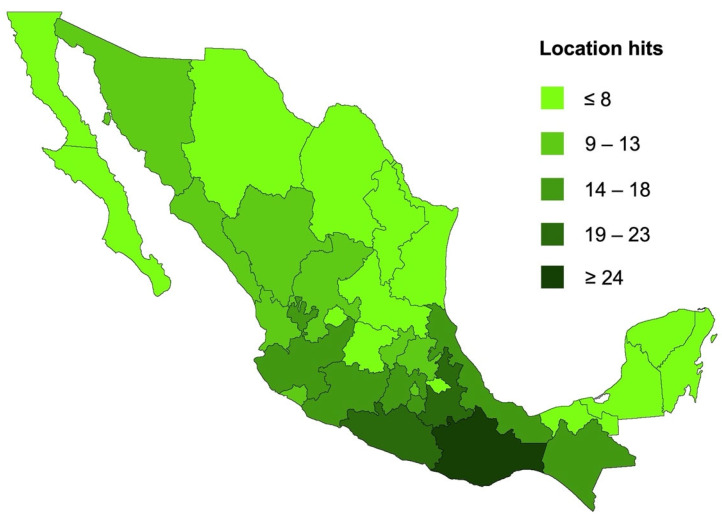
Geographical origin and distribution of Mexican plants as a source of PCTs with bioactivity according to published reports and distribution frequencies.

Different scientific repositories were selected to find reports regarding PCTs from Mexican plants. The terms “pentacyclic triterpenes” AND “Mexican plants” OR “Mexican” OR “Mexico” OR “bioactivity” were used in the search queries, and from the obtained results, research articles that evaluated the biological activity of isolated PCTs or tested extracts containing PCTs during the last 22 years were selected. In several cases, PCT-producing plant species are spread worldwide, but we attempted to focus on the biological diversity present in Mexico. As a result, we found 31 reports of bioactivity studies. The location of the studied plants was established according to the recollection data (when available) and the species’ distribution reported by public organisms and/or scientific sources. So, the plant geographical location by state in these research studies related to 346 absolute hits. The tendencies in the distribution (Figure 3) show that higher hits of the investigated plants are found in the southwest and center of México, which correlates with the high biodiversity of Mexico in this zone.

### 2.1. Biological Activity Survey of PCTs Found in Mexican Plants

**Figure 4:**
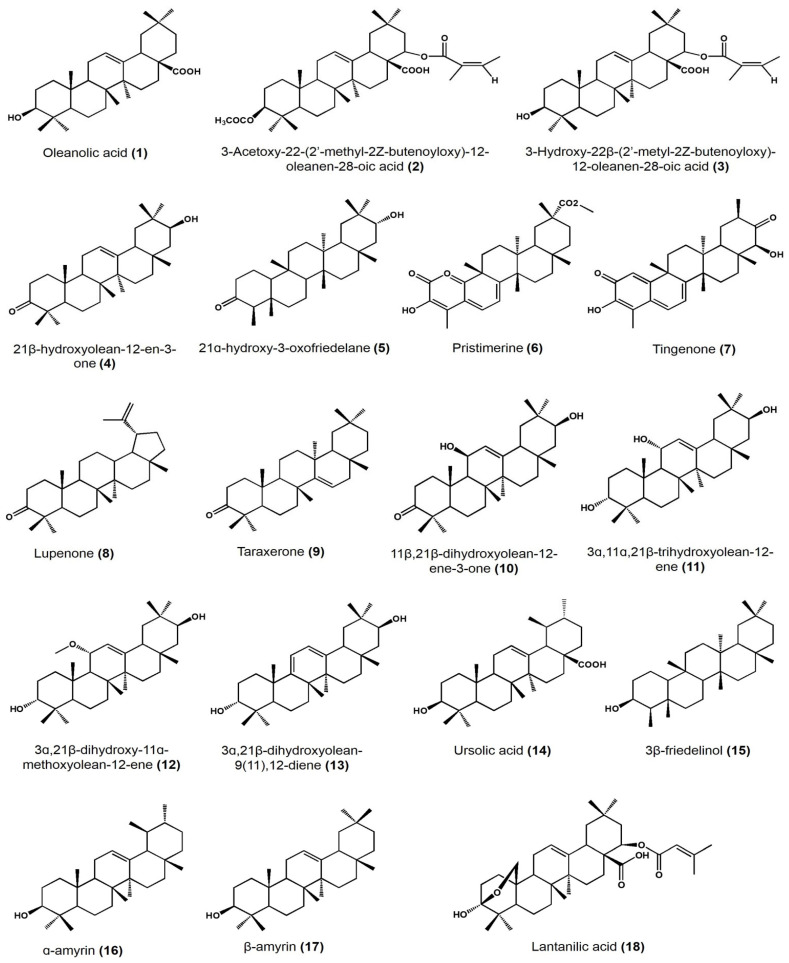

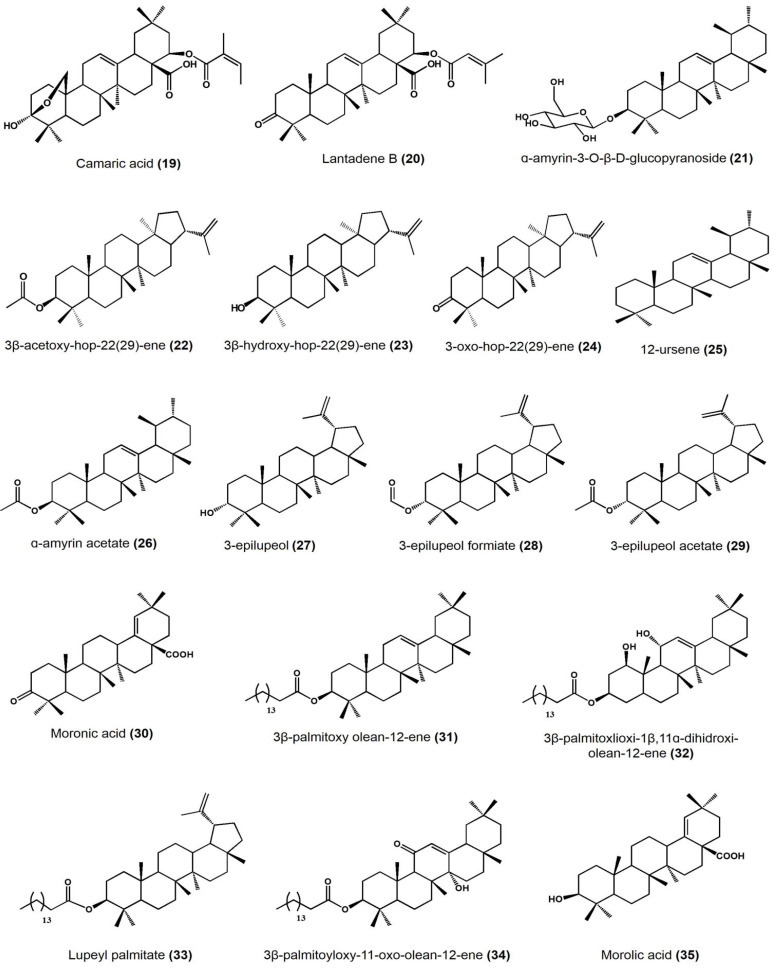

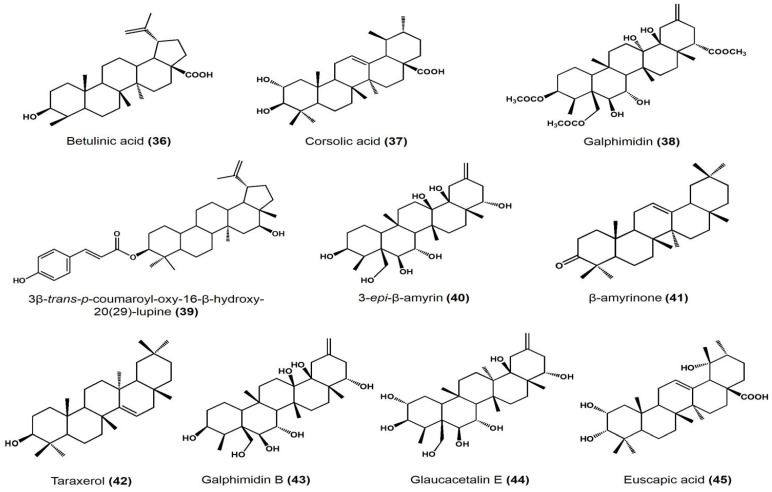
Chemical structures of PCTs identified in native Mexican plants.

A summary of the different reported bioactivities of PCTs found in Mexican plants is presented in Table 1. Figure 4 presents the structure of the PCTs used in the discussion of the following subsections, with the corresponding structure indicated by the bold numbers in parentheses in the next lines.

One of the most evaluated properties is antimicrobial activity. In this regard, the team of Jiménez-Arellanes conducted the extraction of three oleanane-type triterpenoids from *Lantana hispida:* oleanolic acid **(1)** 3-Acetoxy-22-(2′-methyl-2Z-butenoyloxy)-12-oleanen-28-oic acid **(2)**, 3-Hydroxy-22β-(2′-metyl-2Z-butenoyloxy)-12-oleanen-28-oic acid, and **(3)** reduced lantadene A [29]. These compounds were tested at a concentration ranging from 6.25 to 200 µg/mL against *Mycobacterium tuberculosis* H37Rv and its drug-resistant strains, with each one resistant to isoniazid, rifampin, streptomycin, and ethambutol, respectively. As a result, compound **(1)** showed a minimum inhibitory concentration (MIC) equal to 25 μg/mL on the H37Rv, and an MIC of 50 μg/mL when tested on the drug-resistant strains. Compound **(2)** showed an MIC of 25 μg/mL on the strains resistant to streptomycin and isoniazid, and an MIC of 50 μg/mL when tested on the H37Rv strain and those resistant to rifampin and ethambutol. Finally, compound **(3)** showed an MIC of 50 μg/mL against the five strains [29].

Another contribution to this field was performed by Mena-Rejón and collaborators [30]. They isolated 21β-hydroxyolean-12-en-3-one **(4)**, 21α-hydroxy-3-oxofriedelane **(5)**, pristimerine **(6)**, and tingenone **(7)** from the root bark of *Hippocratea excelsa.* The activity against *Giardia intestinalis* was tested at a concentration for each compound in the range from 1.6 to 13.3 μg/mL with an inoculum of 5 × 10^4^ trophozoites/mL, using metronidazole as the positive control. The results showed that compound **(6)** had the greatest activity (IC_50_ 0.11 μM), followed by **(7)** (IC_50_ 0.74 μM), **(5)** (IC_50_19.8 μM), and lastly, **(4)** (IC_50_ 27.4 μM). Compounds **(4)** and **(5)** showed an inhibitory activity greater than metronidazole (IC_50_ 1.23 μM) [30].

In another antimicrobial study, the authors isolated two PCTs from Acacia cochliacantha and tested its activity against *Staphylococcus aureus, Bacillus subtilis, Enterococcus faecium, Lactiplantibacillus plantarum, Escherichia coli, Salmonella typhimurium, Klebsiella pneumoniae,* and *Pseudomonas aeruginosa* [30]. Lupenone **(8)** had the highest MICs (22.5 mg/mL) against *K. pneumoniae* and *P. aeruginosa* while taraxerone **(9)** had a high MIC (2.8 mg/mL) against *P. aeruginosa* [31].

Cáceres-Castillo et al. found five PCTs in *Hippocratea excelsa:* **(4)**, 11β,21β-dihydroxyolean-12-ene-3-one **(10)**, 3α,11α,21β-trihydroxyolean-12-ene **(11)**, 3α,21β-dihydroxy-11α-methoxyolean-12-ene **(12)**, and 3α,21β-dihydroxyolean-9(11),12-diene **(13)** [32]. These compounds were tested in DMSO solution on *Giardia intestinalis* trophozoites in the log phase, except for compound **(4)**, which was previously evaluated by Mena-Rejón et al. [29]. PCT **(13)** had the more significant and highest activity (IC_50_: 78, CI_95_: 77.2–79.1 μM), followed by **(11)** (IC_50_: 96.8, CI_95_: 96.8–99.2 μM), **(10)** (IC_50_: 184.6, CI_95_: 179.8–190.1 μM), and, lastly, **(12)** (IC_50_: 690.7, CI_95_: 650.6–736.9 μM). As a control, metronidazole showed an IC_50_ of 1.2 μM (CI_95_ of 0.88–1.59 μM) [32].

In the same field, the team of Jiménez-Arellanes extracted oleanolic acid **(1)** and ursolic acid **(14)** from *Chamaedora tepejilote* and *Lantana hispida*, respectively, performing distinct bioactivity assays [33]. Firstly, the compounds were tested in vitro individually and as a mixture against the *Mycobacterium tuberculosis* H37Rv strain (multidrug-resistant to isoniazid, rifampicin, ethambutol, and streptomycin) and four strains monoresistant to isoniazid, rifampicin, ethambutol, and streptomycin, respectively. Among all the tested strains, **(14)** had an MIC of 25 μg/mL, except for streptomycin-monoresistant *M. tuberculosis*, for which the MIC was 12.5 μg/mL. On the other hand, **(1)** showed the highest MIC at 50 μg/mL against the multidrug-resistant (H37Rv) and streptomycin-resistant strains. Additionally, both compounds were applied as a mixture, having an MIC of 12.5 μg/mL against the H37Rv strain and an MIC of 25 μg/mL on the other monoresistant strains. As part of this study, both **(1)** and **(14)** were tested against two clinical isolates: MMDO (resistant to isoniazid and ethambutol) and MTY 147 (resistant to isoniazid, rifampicin, ethambutol, and ethionamide), for which **(14)** showed a 25 μg/mL MIC whereas **(1)** showed an MIC of 50 μg/mL [33]. Secondly, five non-tuberculosis *Mycobacterium* species were used for the in vitro activity: *M. chelonae, M. avium, M. fortuitum, M. smegmatis*, and *M. simiae*. Compound **(1)** and the mixture of both acids had an MIC of 100 μg/mL on all species, whereas **(14)** showed the same MIC value on the first three species and 200 μg/mL for the last two species [33].

In addition, the same authors studied the activity of both compounds in a macrophage model considering the H37Rv strain and the MTY 147 clinical isolate [33]. For this purpose, macrophages (cell line J774A.1) were infected with both strains separately and treated with several combined ratios of the compounds. After several incubation times (3, 6, 24, and 48 h), the macrophages were lysed in order to measure the intracellular bacillus colony-forming units (CFU) inside them. After 48 h, the mixture (6.25 **(14)** + 12.5 **(1)** μmol/mL) had the highest reduction count (up to 10^1^ CFU/mL) on the MDR isolate and the H37Rv strain (up in the same way as the mixture of 0.625 **(14)** + 1.25 **(1)** on the H37Rv strain). In this same study, the team infected BALB/c mice with the H37Rv strain, measuring the bacilli load per lung and the percentage of lung surface area affected by pneumonia after a mixture of both compounds was administered. Briefly, after 60 days of infection, 5 mg/kg of each triterpene was dissolved in ultra-pure olive oil and a total volume of 100 μL was administered subcutaneously to the mice (proportion 3:1 of **(14)** and **(1)**) 3 times a week for 30 and 60 days [33]. Over time, a decrement in the bacilli load was observed; after 60 days, the charge of the treated mice was approximately 0.1 × 10^6^ bacilli/lung and near 0.9 × 10^6^ bacilli/lung in the control mice. For the same period, the percentage of lung surface area affected by pneumonia was 11% and 32% for the treated and control mice, respectively. In the same experiment, the levels of the expression of the genes encoding IFN-γ, TNF-α, and iNOS were measured, concluding that animals treated with the mixture exhibited higher mRNA expression of IFN-γ and TNF-α; however, this was not statistically significant when compared to the control, whereas the iNOS mRNA expression was significantly higher than non-treated animals. Finally, the combination of the triterpene mixture with conventional chemotherapy (10 of triterpene mixture with conventional chemotherapy (10 μg/kg of rifampicin, 10 μg/kg isoniazid, and 30 μg/kg pyrazinamide)) was analyzed after 7, 14, 30, and 60 days of treatment. The lowest bacilli load per lung was observed on day 30 (approximately 0.003 × 10^6^ bacilli/lung with respect to 0.005 × 10^6^ bacilli/lung in the control population) in the PCTs mixture plus antibiotics. Instead, the lowest lung surface percentage affected by pneumonia occurred on day 60, with an approximate percentage of 13% with respect to 10% of mice treated only with antibiotics. At the transcriptional level, for IFN-γ, TNF-α, and iNOS, PCTs plus antibiotics induced high expression [33].

PCTs’ antimicrobial activity has also been analyzed by Egas et al., who extracted 3β-friedelinol **(15)** and a mixture of α-amyrin **(16)** and β-amyrin **(17)** from *Heterotheca inuloides* (Mexican arnica) and tested them against *Helicobacter* pylori, employing concentrations ranging from 1.95 to 31.25 μg/mL [34]. In this study, none of the tested concentrations showed a 100% bacterial inhibition of the bacterial growth and due to this reason, testing of the compounds at concentrations higher than 31.25 μg/mL is required to identify their respective MIC.

Lantanilic acid **(18)**, camaric acid **(19)**, and lantadene B **(20)** from *Lantana camara* var. *aculeata* (L.) were isolated and evaluated against *Leishmania mexicana* promastigotes and *L. amazonensis* amastigotes [35]. It is important to mention, prior to the isolation of the noted compounds, that a series of chromatographic fractions were proven. Both **(18)** and **(19)** acids, together with **(20)**, were part of a chromatographic fraction (mixture) that led to an IC_50_ of 7.9 ± 0.3 and 11.2 ± 2.2 μg/mL against *L. amazonensis* and *L. mexicana*, respectively. Additionally, **(18)** and **(19)** in another fraction showed an IC_50_ of 23.6 ± 2.6 and 2.8 ± 0.5 μg/mL with the same species. Additionally, **(20)** was identified in a mixture, with an IC_50_ of 8 ± 1.1 and 22 ± 9.3 μg/mL on *L. amazonensis* and *L. mexicana*, respectively. As control drugs, pentamidine for *L. amazonensis* and Glucantime^®^ (Sanofi-Aventis) for *L. mexicana* were used. In their isolated form, these PCTs had the following IC_50_s against *L. mexicana*: 9.50 ± 0.28 μM for **(18)**, 2.52 ± 0.08 μM for **(19)**, and 23.45 ± 2.15 μM for **(20)** while the reference drug had an IC_50_ of 11,000 ± 2.15 μM. In addition, the mixtures of the compounds presented a cytotoxic effect against BALB/c mouse peritoneal macrophages when tested from 12.5 to 200 μg/mL and a half maximal cytotoxic concentration (CC_50_) higher than 100 μg/mL [35].

Recently, a multi-institutional research group reported cytotoxic and antibacterial activity in *Cisus incisa* leaf extracts due in part to α-amyrin-3-O-β-D-glucopyranoside **(21)** among other phytocompounds [36]. For the antimicrobial testing, the team employed methicillin-resistant *Staphylococcus aureus* (MRSA), linezolid-resistant *Staphylococcus epidermis* (LRSE), vancomycin-resistant *Enterococcus faecium* (VREF), carbapenems-resistant *Acinetobacter baumanii* (CRAB), *Escherichia coli* producing extended-spectrum β-lactamase (ESBL), *Pseudomonas aeruginosa* resistant to carbapenems (PARC), carbapenems-resistant *Klebsiella pneumoniae* NDM-1 + (New Delhi metallo-β-lactamase) (KPNDM-1+), *Klebsiella pneumoniae* producer of ESBL (KPPB), and carbapenems-resistant *Klebsiella pneumoniae* producer of OXA-48 oxacillinase (KPOX). The phytochemicals were applied at 200, 100, 50, 25, 12.5, 6.25, and 3.12 μg/mL, using levofloxacin at the same doses and DMSO as the negative control. This study showed that compound **(21)** had an MIC of 100 μg/mL against PARC; however, all the other tested microorganisms showed an MIC higher than 200 μg/mL [36].

PCTs have also been evaluated as anti-inflammatory agents. López-Huerta et al. reported the presence of three hopane-type triterpenes in *Cnidoscolus spinosus* with anti-inflammatory activity on mouse edema, and with antiparasitic and yeast α-glucosidase inhibitory activities [37]. For the anti-inflammatory profiling, the edema was induced in mice by administrating a solution of 1.25 μg/ear of TPA (12-*O*-tetradecanoylphorbol acetate) after the administration of the tested compounds. Indomethacin was used as the positive control at the same dose, and CHCl_3_ was the negative control. After this first step, the compounds that showed an inhibition percentage greater than 50% were evaluated at a dose range from 0.18 to 1 μmol in 20 μL of chloroform. At a dose of 0.31 μmol/ear, 3β-acetoxy-hop-22(29)-ene **(22)** had the highest inhibitory activity among the three PCTs (57.27 ± 16.99%), followed by 3β-hydroxy-hop-22(29)-ene **(23)** (27.05 ± 7.38%) and 3-oxo-hop-22(29)-ene **(24)** (17.5 ± 4.11%). The authors identified that the most effective PCT **(22)** had a dose-dependent activity, with an ID_50_ of 0.36 μmol/ear, in comparison with indomethacin, the ID_50′_ of which was 0.24 μmol/ear. Regarding the inhibitory activity of α-glucosidase, the authors used a mammalian (rat) (type 2 α-glucosidases) and yeast (type 1 α-glucosidases) model, testing PCTs at 1, 10, and 100 μM. The activity was concentration dependent. The highest inhibition was shown by compound **(22)**, which had an activity of 11.49, 83.5, and 98.79% for doses of 1, 10, and 100 μmol/ear, respectively. In second place, compound **(23)** showed an inhibition of 3.75, 4.41, and 23.47%; and lastly, the PCT **(24)** showed no inhibition at 1 μmol/ear but inhibited the enzyme by 1.51 and 7.39% at the remaining concentrations [37]. In this study, the authors found that the α-glucosidase inhibitory activity was lower in the mammalian test. In addition, the antiparasitic activity against epimastigotes of *Trypanosoma cruzi* Silvio, Cl Brener, and Queretaro strains and *T. rangeli* and *Leishmania mexicana* was measured at a concentration of 50 μM. Compounds **(23)** and **(24)** reduced the viability of the Queretaro strain by more than 20% without affecting the viability of the host (Vero cells). Compound (**24**) was subsequentially tested at 50, 100, 200, and 400 μM, and **(22)** at 50 μM after 24 and 48 h. It is important to note that at 24 h, **(24)** reduced the viability of the Queretaro strain by more than 20% at all tested concentrations in a dose-dependent manner. It also had moderate effects on *T. rangeli* and *L. Mexicana,* but it did not affect the viability of the Vero cells at any of the studied doses. On the other hand, the same PCT **(24)** lost its antiparasitic activity against the *T. cruzi* strain after 48 h, but it started to affect the viability of the Vero cells. However, it showed inhibition on the Cl Brener and Silvio strains and *L. mexicana*. Finally, after 48 h, compound **(23)** did not affect the Vero cells nor the viability of *T. cruzi* [37].

In another study, Perez and Vargas identified the presence of 12-ursene **(25)** in *Agarista mexicana* [38]. They analyzed the effects of the PCT on blood glucose levels in normal and alloxan-diabetic mice. Diabetes was induced by applying 70 mg/kg of alloxan 3 times every third day. Then, the triterpenes were administered intraperitoneally at a concentration of 50 mg/kg. In the normoglycemic mice, the animals were fasted 12 h prior to the experiment, and the blood glucose levels were measured after triterpene administration at 1.5, 2, 4.5, and 24 h [38]. Tolbutamide at 50 mg/kg was applied as a control, since this drug is responsible for stimulating pancreas β-cells, leading to an increase in insulin secretion [46]. In normal mice, the greatest glucose reduction was achieved 4.5 h after the administration of PCT. Compound **(25)** led to a mean reduction of 25.9 ± 6.1%. In a comparable way, in alloxan-induced hyperglycemic mice, the highest decrement in glucose levels was shown after 3 and 4.5 h of administration [38].

The genus Bursera is composed of trees and shrubs and 80 species are distributed widely in Mexico [39]. The resin of *Bursera copallifera,* known as “copal”, yielded six PCTs. Anti-inflammatory in vivo tests were carried out in mice through the induction of edema by applying phorbol ester 12-O-tetradecanoylphorbol-13-acetate (TPA) at a dose of 1, 0.75, 0.50, 0.25, and 0.125 mg/ear, using indomethacin as a positive control at the same doses, and acetone as a negative control [39]. At a dose of 1 mg/ear, the highest inhibition effect was observed with α-amyrin acetate **(26)** (69.45 ± 1.8%), followed by 3-epilupeol **(27)** (66.39 ± 4.38%), 3-epilupeol formiate **(28)** (62.16 ± 1.8%), lupenone **(8)** (57.25 ± 1.36%), 3-epilupeol acetate **(29)** (49.35 ± 3.6%), and, lastly, α-amyrin **(16)** (25 ± 1.81%). The previously mentioned compounds had an ID_50_ of 1.17, 0.83, 0.96, 1.052, >2.13, and >2.34 μmol/ear, respectively. Additionally, the authors measured the effect of the compounds on nitric oxide (NO) production in macrophages (RAW 264.7 cell line). For this purpose, the macrophages were cultured with the tested compounds at 4.37, 8.75, 17.5, 35, and 70 μM, and dimethyl sulfoxide (DMSO) at 0.5% *v/v* or indomethacin at 30 μg/mL were used as the negative and positive controls, respectively. Lipopolysaccharides (LPSs) (10 μg/mL) were added to the macrophages in the presence or absence of the mentioned compounds to incubate the cells and stimulate NO production. Among all the tested compounds, **(28)** had the greatest NO IC_50_ (43.31 ± 2.60 μM), followed by **(29)** (31.13 ± 1.25 μM), (26) (22.47 ± 1.19 μM), **(8)** (20.8 ± 1.07 μM), **(27)** (15.50 ± 1.14), and, lastly, **(16)** (8.98 ± 1.73 μM). In this study, indomethacin showed an IC50 of 54.69 ± 10.34 μM [39].

Figueroa-Suárez and collaborators extracted α-amyrin **(16)**, moronic **(30)**, and ursolic acids **(14)** (Figure 4) from *Bursera cuneata* (Schldl.) Engl aerial parts with dichloromethane [40]. The identified compounds showed significant edema inhibition activity in comparison with indomethacin (positive control) in the ears of a mouse model. For the assay, TPA was applied at a dose of 2.5 µg/ear and a concentration of 0.1 mg of compound/ear. Then, they calculated the edema inhibition percentage by comparing the ear weight before the addition of only TPA versus the addition of TPA plus the treatment. In this part of the study, they identified that **(16)**, **(30)**, and **(14)** showed relevant enema inhibition (44.9 ± 1.2, 68.1 ± 1.3, and 55.6 ± 2.1%, respectively), in comparison with indomethacin (41.5 ± 0.8% at the same dose), an inhibition percentage lower than the three pentacyclic triterpenes studied. These edema inhibition percentages correlated with the histamine inhibition results, with **(30)** being the compound with the major activity (73.3 ± 1.1%). It is important to mention that the anti-inflammatory effect **(30)** was dose dependent, evaluating doses of 0.03, 0.1, 0.3, and 0.5 mg/ear. Additionally, the authors studied the effect of **(30)** on macrophage viability using RAW 264.7 cells at a concentration ranging from 0.3 to 60 µg/mL or with the control etoposide at 20 µM for 24 h. As result, at 60 µg/mL, the viability of the macrophages was reduced by 43% [40].

In a similar way, another research group identified four PCTs in the organic extract of *Sapium lateriflorum* and analyzed their anti-inflammatory and cytotoxic activities [41]. For the latter, the team used six human tumor cell lines: U251 (glioblastoma), PC-3 (prostate), K562 (leukemia), HCT-15 (colon), MCF-7 (breast), and SKLU-1 (lung) at doses of 50 μg/mL using etoposide as the positive control. 3β-palmitoxy olean-12-ene **(31)** showed a 1.81% (PC-3), 6.88% (K562), 10.46% (HCT-15), 3.24% (MCF-7), and 21.95% (SKLU-1) inhibitory effect while 3β-palmitoxlioxi-1β,11α-dihidroxi-olean-12-ene **(32)** generated a 29.56% (U251), 20.72% (PC-3), 9.86% (K562), 7.24% (HCT-15), 11.53% (MCF-7), and 12.89% (SKLU-1) inhibition. Moreover, using lupeyl palmitate **(33)**, the cell lines showed an inhibition of 3.05% (PC-3), 3.46% (K562), 10.24% (HCT-15), 13.43% (MCF-7), and 19.59% (SKLU-1). Finally, 3β-palmitoyloxy-11-oxo-olean-12-ene **(34)** generated an inhibition of 0.08% (PC-3), 18.69% (HCT-15), 5.15% (MCF-7), and 20.06% (SKLU-1). In comparison, etoposide had an inhibition of 91.1% (U251), 51.4% (PC-3), 60.2% (K562), 80.8% (HCT-15), 56.8% (MCF-7), and 81.7% (SKLU-1) [42]. For the anti-inflammatory evaluation, the team induced mouse ear edema by applying TPA and then triterpene or indomethacin (positive control). PCT **(32)** showed an inhibition of 68.76% while **(31)** had a 48.26% effect, followed by **(33)** and **(34)**, with an inhibition of 22.31% and 31.49%, respectively, while indomethacin had a 78.76% inhibition. Due to the high activity of **(32)**, the team measured its ID_50_ on mouse edema, showing a value of 0.60 μmol/ear. On the other hand, indomethacin had an ID_50_ of 0.24 μmol/ear. Compound **(32)** was also evaluated on the carrageenan-induced mouse plantar edema model, using a dose of 31.6 mg/kg and reaching the peak of the inhibitory activity after 3 h (48.2%) in a similar way to indomethacin (7.5 mg/kg dose, 74.1% inhibition after 3 h). The activity of myeloperoxidase (MPO) was determined to study the effects of **(32)** on the inflammatory process. After the addition of TPA to the mouse ear, each compound was administrated from 0.1 to 1.78 μmol/ear, showing the highest reduction in MPO activity when the compound was applied at 1 μmol/ear [41].

In addition to the previously noted cases, the roles of PCTs have also been explored on assays regarding their vasorelaxant effects. In 2012, Rios et al. extracted four PCTs (ursolic **(14)**, moronic **(30)**, morolic **(35)**, and betulinic **(36)** acids) from *Phoradendron reichenbachianum* (Viscaceae) to profile its vasorelaxant activity [42]. In this case, the team used rat aorta rings (present endothelium) between two metallic hooks to measure the contractile force (% of relaxation) changes after the addition of the compounds and NA (noradrenaline bitartrate) for pre-contracting the tissues before the addition the PCTs. The highest maximum effect (E_max_) was observed in **(30)** (92.01%, EC_50_ (half maximal effective concentration) 11.7 µM), followed by **(36)** (79.01%, EC_50_ 58.46 µM), **(35)** (73.75%, EC_50_ 94.19 µM), and, lastly, **(14)** (72.59%, EC_50_ 11.7 µM) [42]. The mentioned data belongs to procedures where endothelium was present in the aortic rings.

More recently, three different PCTs (corsolic acid **(37)** from *Cratageus gracilior*, galphimidin **(38)** from *Galphimia* glauca, and 3β-*trans-p*-coumaroyl-oxy-16-β-hydroxy-20(29)-lupene **(39)** from *Jatropha neopaucilfora*) were investigated for their vasorelaxant activity [43]. These compounds had been previously extracted and isolated by the same team. In this study, L-phenylephrine stimulated the contraction of rat aortic rings before the addition of the compounds. Acetylcholine was used as a positive control, with 58.8 ± 8.9 μM as EC_50_ and 69.5 ± 5.7% as E_max_. Regarding the results, **(37)** had a significant effect (EC_50_ 108.9 ± 6.7 μM, E_max_ 96.4 ± 4.2%) compared to the other two PCTs: **(38)** showed an EC_50_ equal to 145.9 ± 6.7 μM, and an E_max_ of 99.5 ± 5.3% while **(39)** had values of 63.2 ± 5.8 μM and 27.5 ± 1.9% for EC_50_ and E_max_, respectively. It is important to note that **(37)** and **(38)** had a higher E_max_ than the positive control while **(39)** showed an EC_50_ closer to the positive control than the other two PCTs [43].

Although the biological activities of PCTs have been essentially evaluated with microorganisms or mammals, plants have been considered too. In this way, Macías-Ruvalcaba and collaborators carried out trials investigating the effects of six PCTs from *Sebastiania adenophora* on the root growth of amaranth (*Amaranthus hypochondriacus* L.), tomato (*Lycopersicon esculentum* Mill. var. Pomodoro), and barnyard grass (*Echinochloa crus-galli* L. Beauv) [44]. The isolated compounds were 3-*epi*-β-amyrin **(40)**, β-amyrinone **(41)**, 3-*epi*-lupeol **(27)**, lupenone **(8)**, taraxerol **(42)**, and taraxenone **(9)**. As part of the results, it was found out that all PCTs had a negative effect on the root growth of tomato and barnyard grass when these were applied at a dose of 250 μg/mL. Of all the plant–PCT combinations, **(42)** had the greatest root growth inhibition effect on barnyard grass, with a 77% inhibition, which demonstrates its potential as an allelopathic substance. On the other hand, at the same dose, the tested compounds favored the root development of amaranth. Particularly, **(40)** was the strongest stimuli (55%) of root growth. In addition, the authors tested how the applied dose was related to the effects of **(42)**, **(40)**, and the mixture of the six PCTs when applied at 125, 250, and 500 μg/mL. In amaranth, the effect was dose dependent on all the tested compounds. However, in barnyard grass, the mixture of the six PCTs had a root-stimulating effect when the dose was 125 μg/mL and an inhibitory effect when the doses were higher. A similar behavior was observed in tomato with **(40) [44]**.

Additionally, Rios et al. indicated the presence of three nor-triterpenes in *Galphimia glauca:* Galphimidin **(38)**, galphimidin B **(43)**, and glaucacetalin E **(44)** [45]. The compounds were extracted from aerial parts using methanol as the solvent. Anxiolytic effects were detected in mice after the administration of the compounds mentioned. Galphimidin had the strongest anxiolytic effects of the three compounds, which can be quantitatively compared with those of diazepam. It was observed that the response depended on the administered dose (1, 10, and 30 mg/kg) and the extract had a sedative effect, with a value greater than diazepam [45].

There are some works in which no previously isolated compounds were evaluated in bioassays; instead, extracts containing PCTs were studied. In these cases, the presence of PCTs is known by previous reports that achieved their characterization. There are also other studies in which triterpenes were used as part of a partially purified mixture. Oleanolic **(1)** and ursolic **(14)** acids together with β-sitosterol and stigmasterol (sterols) as the first two fractions in a primary chromatography from an ethanolic extract of *Moussonia deppeana* showed anti-inflammatory activity by inducing edema in the ear of Balb/C mice in two models: TPA and carrageenan [47]. In the first model, fractions were applied at a dose of 2 mg/ear and 2.5 μg of TPA was applied 30 min later. The positive control, indomethacin, was used at 0.5, 1, and 2 mg/ear. After 6 h, for the first fraction, the inhibition was 31.24% in males and 27.24% in females; and for the second fraction, the males showed an inhibition of 53.03% and the females showed an inhibition of 27.24%. For the carrageenan model, the mice received carrageenan at 2% to induce paw edema. After 1 h of application, the animals received each mixture separately at a dose of 150 mg/kg. After 5 h, fraction one showed inhibitory activity of 11.31% and 19.06% for male and female mice, respectively. Finally, fraction two generated an inhibition of 3.70% and 19.84% in both respective sexes [47].

Another example of the evaluated extracts is the research performed by Sánchez-Monroy et al., who prepared dichloromethane-soluble extracts from multiple Mexican *Bursera* species (*Bursera lancifolia, B. fagaroides, B. grandifolia, B. longipes, B. morelensis, B. bicolor*, and *B. submoleniformis)* and tested them on seven cell lines [48]. Regarding the results, the *Bursera bicolor* species caused high growth inhibition in colorectal (HCT-15) and lung (SK-LU1) cancer cell lines, while *B. fagaroides* was active against human glioblastoma astrocytoma (U-251), prostate adenocarcinoma (PC-3), chronic myelogenous leukemia (K-562), colorectal adenocarcinoma, and lung adenocarcinoma (SK-LU1). However, it also showed action against normal fibroblasts, whereas *B. bicolor* extracts did not. In general, the lung cell line was the most susceptible to the evaluated extracts, even with those of *B. grandifolia, B. morelensis*, and *B. linanoe*. In this case, the triterpenes determined were lupenone **(8)**, α-amyrin **(16)**, 3-epilupeol **(27)**, and sitosterol in these dichloromethane-soluble bark extracts of all *Bursera* tested species [48]. It is important to clarify that the authors attributed the observed activity in the extracts to the PCTs; however, the extracts also contain lignans (deoxypodophillotoxin and acetylpodophillotoxin), podophyllotoxin-type lignans with proven cytotoxic activity, and some fatty acids according to Khaled et al. [49]. Hence, the anticancerogenic activity may be a synergy of all components in the prepared extracts. Additional experiments are necessary to identify the individual contributions of the compounds to the cytotoxicity of all tested cancer cell lines.

Additionally, methanolic extract of flowers from *Crataegeus gracilior* presented vasorelaxant effects on rat aortic rings [50]. The extract had an EC_50_ of 1.83 ± 1.39 μg/mL and an E_max_ of 100 ± 3.4%, being 5-fold more potent than the positive control acetylcholine (ACh) (EC_50_ of 9.77 ± 0.81 μg/mL and an E_max_ of 66.72 ± 1.07%). In this study, the team identified corsolic **(37)** and euscapic **(45)** acids as part of the main components in the extract and established the hypothesis of these two components as being responsible for the vasorelaxant effects.

### 2.2. Extraction and Purification Methods

According to the reviewed literature, the initial step for PCT purification is material preparation. PCTs can be found in the aerial parts, stems, and resin of the identified plants, which must be dried and milled prior to the extraction procedures. Usually, for extraction, organic solvents are used for the extract preparation as binary mixtures, dichloromethane (CH_2_Cl_2_), hexane (C_6_H_14_), chloroform (CHCl_3_), and methyl alcohol (CH_3_OH) are the most adopted solvents [31,35,36,37,38,41,50]. The main extraction techniques used for PCTs from Mexican plants are maceration and Soxhlet extraction [30,37,39,47]. Maceration generally occurs during a period ranging from 24 h to 9 days. It has also been reported that the temperature of the system must be below 40 °C [29,35,40]. Once the maceration is completed, the mixture can be filtered and then the solvents must be completely or partially removed under reduced pressure conditions. In some cases, the maceration-extraction procedure can be carried out in multiple steps, as reported by López-Huerta et al. [37].

Once the organic extract is prepared, the compounds are fractionated through chromatographic columns (CCs) using silica gel as the stationary phase [29,31,32,33,34,35,36,37,38,39,40,41,42,43,44,45], and hexane, ethyl acetate, acetone, and di- and tri- chloromethane are the most common solvents used for the mobile phase [34,37,39,40]. First, the extract is processed through the CC and when it is required, these fractions are pooled. In the analysis of the solvent systems used in the elution of PCTs through silica gel chromatography, it is notable that the solvent mixtures present estimated dielectric constant values [51] ranging from 4.0 to 20.0 (Table S1), showing that PCTs have a non-polar to slightly polar character, since solvents with a dielectric constant lower than 15 are generally considered non-polar [51]. This nature is highly dependent on the substituents in the PCT. For example, for non-polar compounds such as galphimidins **(38, 43, 44)**, mixtures contain n-hexane with dichloremetane, acetone, and ethyl acetate [45] while for more polar compounds such as oleanolic **(1)**, ursolic **(14),** and their derivates **(2, 3)**, systems involving hexane-methanol and dichlorometane-methanol are used [29,33]. Some fractions can be processed again in a second CC to increase the compound purity [29,36,37].

In most cases, during CC, the fractions are analyzed by thin-layer chromatography (TLC) [29,30,32,33,34,35,36,37,38,39,40,41,42,43,44,45], where spraying of a solution of 1% (NH_4_)_4_Ce(SO_4_)⋅2H_2_O reveals the triterpene [40,42]. Additionally, in TLC visualization, solutions of oleum reagent [30,32], sulfuric acid 10% or anisaldehyde 10% plus glacial acetic acid 5% plus 5% H_2_SO_4_ [29], H_2_SO_4_ 10% [33], charring solution [34], Liebermann–Burchard’s reagent [35], ceric sulfate in sulfuric acid [36], cerium sulfate (0.1% CeSO_4_/2 N H_2_SO_4_) or vanillin (1% vanillin/5% H_2_SO_4_) [43], or 1% solution of (NH_4_)_4_Ce(SO_4_)_4_ in 2N H_2_SO_4_ [42] have been applied too.

For analytical purposes, HPLC [34,43] or UHPLC [36] coupled to different detection systems (mass spectrometry, UV, diode-array, evaporative light scattering detector (ELSD)) and gas chromatography [35] have been reported. Nuclear magnetic resonance (NMR) [29,39,40,41,43,44,45], infrared (IR) [29,30,31,32,33,34,36,37,38,41,42,43,44,45], UV [30,32,34,37,42,43,44] and Fourier transform infrared spectroscopy (FTIR) [36], mass spectrometry [30,31,32,34,37,38,41,42,43,44,45], optical rotation measurement [30,31,32,34,37,41,42,43,44], and X-ray diffraction [42] were used for compound identification.

### 2.3. Drug Delivery Strategies

As indicated previously, PCTs have shown a potential therapeutic effect. Nevertheless, this application has not been further exploited since many of these compounds have been recently discovered, and partially because of their disadvantageous properties such as low solubility, stability, and bioavailability. The latter limits intestinal absorption from oral administration [52,53]; hence, adequate drug delivery systems for improving their assimilation in the organism must be developed. In this aspect, the formulations employed for PCT delivery until now are liposomes, emulsions, nanoparticles, and cyclodextrin–drug complexes. In most cases, these have been used with successful results. In a minor degree, dendrimers, capsules, and tablets have been used too [52]. All of them are applicable to diverse administration routes (e.g., oral, intraperitoneal, transdermal, or intravenous). The development, characterization, and evaluation of novel formulations for PCTs, in particular for cancer treatment, have been assayed with oleanolic **(1)**, ursolic **(14)**, and betulinic **(36)** acids, as representative triterpenoids. In vivo studies have shown that formulated PCTs presented better efficiencies than free-form triterpenes and in human preclinical reports, UA liposomes with **(14)** have shown good tolerability and action [53]. In Mexico, scarce work has been carried out that is related to the formulation of PCTs. Two efforts in this aspect include collaborations with other countries and belong to the Chemical Faculty of the Universidad Autónoma del Estado de Morelos with Spanish and Portuguese research groups. In one of these reports, the team formulated nanoemulsions of **(1)** and **(14)** (0.2%) for its topic administration while in the second report, polymeric nanoparticles were loaded with the same PCTs [54,55]. Nanoemulsions consisted of castor oil as the non-polar phase, labrasol as surfactant, transcutol-P as co-surfactant, and propylene glycol in the polar phase together with **(1)** and **(14)**, which were obtained from *Plumera obtusa* leaves, an ornamental tree collected from Campeche, Mexico. The formulation based on this technology is oriented to offer local non-systemic effects since the retention was higher than the permeation, and the inflammation in the mouse ear was inhibited by 76.19% [54]. The poly (DL-lactide-coglycolide) acid (PLGA) nanoparticles containing the PCTs were elaborated by the solvent displacement method, showing good stability and anti-inflammatory activity in rabbit eyes [55]. More recently, in a third study, Calderón-Chiu and collaborators at Tecnologico Nacional de Tepic, Nayarit and the State University of West Paraná in Brazil proposed a nanoemulsion loaded with *Coccoloba uvifera* (sea grape) leaf extract from Veracruz rich in lupeol, α y β-amirin [56]. The ultrasound-formed nanoemulsion was stabilized with 1.25% of jackfruit leaf protein hydrolysate, with the intention of being used in the food industry.

## 3. Opportunities, Limitations, and Future of Pentacyclic Triterpene Research in Mexico

In the present review and analysis of the existing research about PCTs in Mexico, several conclusions and reflections were obtained. Derived from Mexican biodiversity, PCTs belonging to the oleanane group are mainly found in Mexican flora. According to the analyzed information, the greatest area where this class of compounds has been found is the southwest of Mexico, which is related to the richness and diversity in this zone. In this sense, many reports agree with the potential of PCTs (both at national and international levels) as antimicrobial agents and as a therapeutic option to deal with metabolic disorders such as diabetes and hypertension [7] or severe diseases such as cancer [4]. In research matters, PCTs have mostly been assayed for their antimicrobial activity in in vitro studies, but some reports of in vivo studies for the detection of anti-inflammatory effects were also found. In the same way, identification, characterization, and elucidation structure studies have limited access but in a more minor grade than biological activity testing.

These observed trends are associated with the infrastructure and resources available in Mexican research centers where experimentation was carried out. In general, national institutions lack the equipment and budget for bioassays or characterization because these activities require special facilities and maintenance. Since only a few academic institutions own some of these facilities, many collaborations with these universities can be identified in the reviewed works. This limited national infrastructure explains the scarcity of testing for anticancerogenic activity when compared to other observed international efforts.

Drug delivery formulation of these compounds has practically been null in Mexico, as observed in the corresponding section, and international collaborations have made the generation of PCT emulsions possible. In the same way, this behavior is related in part to the equipment deficiencies mentioned previously but also with an abandonment of this research line at a national level and more closely to the current national pharmaceutical policy (regulation and centralization). This last aspect is exemplified with the reported 29% acceptance rate of new drug applications in 2018 by social security institutions [57]. An alternative for encouraging drug delivery development in the future may be the search for synergies with the local industry, public health institutions, and government.

An area of opportunity in the extraction and purification field of PCTs in Mexico is the implementation of novel and current sustainable green techniques (i.e., supercritical fluid extraction, microwave-assisted extraction, ultrasound-assisted extraction, pressurized liquid extraction) coupled with the use of green solvents (i.e., deep eutectic, ionic, water, etc.) [58], with the goals of more environmentally friendly and sustainable processes, energy savings, and reduced formation of toxic effluents [59]. This “green process vision” not only would have an acceptable ecological impact but would also stimulate the interest of the private sector to increase investments and consequently the facilities in research centers. Some researchers have started to take this approach, as in pristimerin recovery with the use of green solvents [60] and the evaluation of process “greenness” [61]; however, the focus is incipient.

Undoubtedly, as mentioned in the introduction, there are still a wide number of Mexican plants that have not been studied and may contain novel or already reported PCTs. Plants are a valuable natural resource for treating diseases that affect every person around the world, solving problems from a social perspective and, at the same time, reaffirming and highlighting part of the cultural heritage of our country.

In this way, the exploitation of PCTs from Mexican plants requires the construction of databases to collect traditional knowledge, which may also be an opportunity for fast identification of these plants. A clear limitation of this research is again the low budget allocations (observed indirectly in the present review as facilities or equipment shortages). Solutions to deal with this hurdle include the attraction of funding and the search for national and international cooperation.

On the other hand, the presence of human resources in centers and universities is one of the points in favor of Mexican research on natural products and of course for PCT research. In this context, leading national groups in natural product research are identified in institutions such as Universidad Autómoma de México, Instituto Politécnico Nacional, Instituto de Ecología, Universidad Autónoma de Nuevo León, Universidad Autónoma de Querétaro, Instituto Tecnológico y de Estudios Superiores de Monterrey, Universidad Autónoma Metropolitana, Centro de Investigaciones Biológicas del Noreste, Colegio de Postgraduados, Instituto Mexicano del Seguro Social, Universidad Autónoma del Estado de Morelos, and Universidad Michoacana de San Nicolás Hidalgo [62]. So, highly trained scientists and researchers may join efforts to achieve interesting outcomes, since, as it can be deduced, this is an integrative field.

## 4. Conclusions

In summary, PCTs are bioactive compounds mainly biosynthesized in diverse plants. About 45 PCTs from 33 Mexican plants have been identified and evaluated to have biological activity, mainly antimicrobial and cytotoxic effects. Bioprocessing to obtain these PCTs has involved plant pretreatment, essentially organic extraction by maceration and Soxhlet and silica column chromatography. Although there is still work pending on the drug delivery and formulation of these PCTs, this research line is promising for providing future alternatives in the treatment of serious diseases in Mexico and other parts of the world. Nevertheless, challenges in terms of the economic resources, equipment and facilities, traceability of these plant sources, and extraction sustainability need to be considered. National and international groups and institutions working with natural products might collaborate with their facilities, human resources, and experience to tackle these challenges in the future.

## Figures and Tables

**Table 1:** PCTs isolated from Mexican plants with biological activity.

Plant Species	Identified Compounds	Subgroup	Activities	References
*Lantana hispida*	Oleanolic acid	Oleanane	Antimicrobial activity (MIC) on *Mycobacterium tuberculosis*: • H37Rv: 25 μg/mL • STR-R, RIF-R, INH-R, and EMB-R: 50 μg/mL	[29]
	3-acetoxy-22-(2′-methyl-2Z-butenyloxy)-12-oleanene	Oleanane	Antimicrobial activity (MIC) on *M. tuberculosis*: • H37Rv, STR-R, and INH-R: 25 μg/mL • RIF-R and EMB-R: 50 μg/mL	
	3-hydroxy-22-(2′-methyl-2Z-butenoyloxy)-12-oleanen-28-oic acid (reduced lantadene A)	Oleanane	Antimicrobial activity (MIC) on *M. tuberculosis*: • H37Rv, STR-R, RIF-R, INH-R, and EMB-R: 50 μg/mL	
*Hippocratea excelsa*	21β-hydroxyolean-12-en-3-one	Oleanane	Antiprotozoal activity (IC_50_) on *Giardia intestinalis*: 27.4 μM	[30]
	21α-hydroxy-3-oxofriedelane	Friedelane	Antiprotozoal activity (IC_50_) on *G. intestinalis*: 19.8 μM	
	Pristimerine	Friedelane	Antiprotozoal activity (IC_50_) on *G. intestinalis*: 0.11 μM	
	Tingenone	Friedelane	Antiprotozoal activity (IC_50_) on *G. intestinalis*: 0.74 μM	
*Acacia cochliacantha*	Lupenone	Lupane	Antimicrobial activity (MIC) on: • *Staphylococcus aureus*: 2.8 mg/mL • *Bacillus subtilis*: 1.4 mg/mL • *Enterococcus faecium*: 5.6 mg/mL • *Lactobacillus plantarum*: 11.3 mg/mL • *Escherichia coli*: 2.8 mg/mL • *Salmonella typhirium*: 22.5 mg/mL • *Klebsiella pneumoniae*: 22.5 mg/mL	[31]
	Taraxenone	Taraxerane	Antimicrobial activity (MIC) on: • *S. aureus*: 0.4 mg/mL • *B. subtilis*: 1.4 mg/mL • *L. plantarum*: 1.4 mg/mL • *E. coli*: 0.2 mg/mL • *S. typhirium*: 2.8 mg/mL • *K. pneumoniae*: 1.4 mg/mL • *Pseudomonas aeruginosa*: 2.8 mg/mL	
*Hippocratea excelsa*	11β,21β-dihydroxy-olean-12-ene-3-one	Oleanane	Antiprotozoal activity on *Giardia intestinalis*: IC_50_: 184.6 μM	[32]
	3α,11α,21β-trihydroxy-olean-12-ene	Oleanane	Antiprotozoal activity on *G. intestinalis*: IC_50_: 96.8 μM	
	3α,21β-dihydroxy-11α-methoxy-olean-12-ene	Oleanane	Antiprotozoal activity on *G. intestinalis*: IC_50_: 690.7 μM	
	3α,21β-dihydroxy-olean-9(11),12-diene	Oleanane	Antiprotozoal activity on *G. intestinalis*: IC_50_:78 μM	
*Chamaedora tepejiliote*	Ursolic acid	Ursane	Antimicrobial activity (MIC) against *M. tuberculosis*: • H37Rv, INH-R, RIF-R, EMB-R, MMDO, and MTY147: 25 μg/mL • STR-R: 12.5 μg/mL In vitro intracellular load of *M. tubrculosis* MDR and H37Rv in macrophages at: • 6.25 μm/mL **(14)** + 12.5 μm/mL **(1)**: (after 48 h) approximately 10^1^ CFU/mL (both strains) • 0.625 μm/mL **(14)** + 1.25 μm/mL **(1**): (after 48 h) approximately 10^1^ CFU/mL (H37Rv) Bacilli load per lung in mice model after 60 days of administration of 1:3 mixture of **(1)**:**(14)** at 5 mg/kg • 0.1 × 10^6^ bacilli/lung approximately • Control: approximately 0.9 × 10^6^ bacilli/lung	[33]
*Lantana hispida*	Oleanolic acid	Oleanane	Antimicrobial activity (MIC) against *M. tuberculosis*: • H37Rv, STR-R, MMDO, and MTY147: 50 μg/mL • INH-R, RIF-R and EMB-R: 25 μg/mL In vitro intracellular load of *M. tubrculosis* MDR and H37Rv in macrophages at: • 6.25 μm/mL: (after 24 and 48 h) approximately 10^5^ CFU/mL. • 0.625 μm/mL: (after 48 h) approximately 10^5^ CFU/mL • Control: approximately 10^6^ CFU/mL Bacilli load per lung in mice model after 60 days of administration of 1:3 mixture of **(1)**:**(14)** at 5 mg/kg • 0.1 × 10^6^ bacilli/lung approximately• Control: approximately 0.9 × 10^6^ bacilli/lung	
*Heterotheca inuloides*	3β-friedelinol	Friedelane	Antimicrobial activity (MIC) on *Helicobacter pylori*: >31.25 μg/mL	[34]
	α-amyrin and β-amyrin	Ursane		
*Lantana camara*	Lantanilic acid	Lantadene	Lehismaniacidal activity on *L. mexicana*: 9.50 ± 0.28 μM	[35]
	*Camaric acid*	Lantadene	Lehismaniacidal activity on *L. mexicana*: 2.52 ± 0.08 μM	
	Lantadene B	Lantadene	Lehismaniacidal activity on *L. mexicana*: 23.45 ± 2.15 μM	
*Cisus incisa*	α-amyrin-3-O-β-D-gluco pyranoside	Ursane	Antimicrobial activity (MIC) against carbapenems-resistant *P. aeruginosa*: 100 μg/mL	[36]
*Cnidosculus spinosus*	3 β-acetoxy-hop-22(29)-ene	Hopane	Inhibitory activity on mouse edema at 0.31 μmol/ear: 57.27 ± 16.99% Inhibition of yeast α-glucosidase at 100 μM: 98.79%	[37]
	3β-hydroxy-hop-22(29)-ene [Hopenol B]	Hopane	Inhibitory activity on mouse edema at 0.31 μmol/ear: 27.05 ± 7.38% Inhibition of yeast α-glucosidase at 100 μM: 23.47% Antiparasitic activity on *Trypanosoma cruzi* at 50 μM: <20%	
	3-oxo-hop-22(29)-ene	Hopane	Inhibitory activity on mouse edema at 0.31 μmol/ear: 17.5 ± 4.11% Inhibition of yeast α-glucosidase at 100 μM:7.39% Antiparasitic activity on Trypanosoma cruzi at 50 μM: <20%	
*Agarista mexicana*	12-ursene	Ursane	Hypoglycemic activity on mice models after at 50 mg/kg: • 25.9 ± 6.1% glucose reduction	[38]
*Bursera copallifera*	lupenone	Lupane	Anti-inflammatory activity in mouse edema inhibition at 1 mg/ear: 57.25 ± 1.36%, ID_50_: 1.052 μmol/ear Reduces NO production in LPS-stimulated macrophages: IC_50_: 20.08 ± 1.07 μM	[39]
*Bursera copallifera*	α-amyrin	Ursane	Anti-inflammatory activity in mouse edema inhibition at 1 mg/ear: 25 ± 1.81%, ID_50_: >2.34 μmol/ear Reduces NO production in LPS-stimulated macrophages: IC_50_: 8.98 ± 1.73 μM	[39]
	α-amyrin acetate	Ursane	Anti-inflammatory activity in mouse edema inhibition at 1 mg/ear: 69.45 ± 1.8%, ID_50_: 1.17 μmol/ear Reduces NO production in LPS-stimulated macrophages: IC_50_: 22.47 ± 1.19 μM	
	3-epilupeol	Lupane	Anti-inflammatory activity in mouse edema inhibition at 1 mg/ear: 66.39 ± 4.38%, ID_50_: 0.83 μmol/ear Reduces NO production in LPS-stimulated macrophages 15.50 ± 1.14 μM	
	3-epilupeol formiate	Lupane	Anti-inflammatory activity in mouse edema inhibition at 1 mg/ear: 62.16 ± 1.8%, ID_50_: 0.96 μmol/ear Reduces NO production in LPS-stimulated macrophages 43.31 ± 2.60 μM	
	3-epilupeol acetate	Lupane	Anti-inflammatory activity in mouse edema inhibition at 1 mg/ear: 49.35 ± 3.6%, ID_50_: >2.13 μmol/ear Reduces NO production in LPS-stimulated macrophages: IC_50_: 31.13 ± 1.25 μM	
*Bursera cuneata*	α-amyrin	Ursane	Anti-inflammatory activity in mouse edema inhibition at 0.1 mg/ear: 44.9 ± 1.2%	[40]
	Moronic acid	Oleanane	Anti-inflammatory activity in mouse edema inhibition at 0.1 mg/ear: 68.1 ± 1.3% Histamine inhibition in a mouse model at 0.1 mg/ear: 73.3 ± 1.1% Macrophages viability reduction at 60 μg/mL: 43%	
	Ursolic acid	Ursane	Anti-inflammatory activity in mouse edema inhibition at 0.1 mg/ear: 55.6 ± 2.1%	
*Sapium lateriflorum*	3β-palmitoyloxy olean-12-ene	Oleanane	In vitro cytotoxicity: inhibitory effect (%) at 50 μM • PC-3: 1.81 • K562: 6.88 • HCT-15: 10.46 •MCF-7: 3.24 • SKLU-1: 21.95 Anti-inflammatory activity in mouse edema inhibition at 1 μmol/ear: 48.26%	[41]
*Sapium lateriflorum*	3β-palmitoxlioxi-1β,11α-dihydroxi-olean-12-ene	Oleanane	In vitro cytotoxicity: inhibitory effect (%) at 50 μM • U251: 29.56 • PC-3: 20.72 • K562: 9.86 • HCT-15: 7.24 • MCF-7: 11.53 • SKLU-1: 12.89 Anti-inflammatory activity in mouse edema inhibition at 1 μmol/ear: 68.76%	[41]
	lupeyl palmitate	Lupane	In vitro cytotoxicity: inhibitory effect (%) at 50 μM • PC-3: 3.05 • K562: 3.46 • HCT-15: 10.24 •MCF-7: 13.43 • SKLU-1: 19.59 Anti-inflammatory activity in mouse edema inhibition at 1 μmol/ear: 22.31%	
	3β-palmitoyloxy-11-oxo- olean-12-ene	Oleanane	In vitro cytotoxicity: inhibitory effect (%) at 50 μM • PC-3: 0.08 • HCT-15: 18.69 •MCF-7: 5.15 • SKLU-1: 20.06 Anti-inflammatory activity in mouse ear edema inhibition at 1 μmol/ear: 31.49%, ID_50_: 0.60 μmol/ear Anti-inflammatory activity in carrageenan-induced mouse plantar edema at 31.6 mg/kg: 48.2% after 3 h	
*Phoradendron reichenbachianum (Viscaceae)*	Ursolic acid	Ursane	Vasorelaxant effect on rat aorta: EC_50_ 11.7 μM, E_max_ 72.59%	[42]
	Moronic acid	Oleanane	Vasorelaxant effect on rat aorta: EC_50_ 11.7 μM, E_max_ 92.01%	
	Morolic acid	Oleanane	Vasorelaxant effect on rat aorta: EC_50_ 94.19 μM, E_max_ 73.75%	
	Betulinic acid	Lupane	Vasorelaxant effect on rat aorta: EC_50_ 58.46 μM, E_max_ 79.01%	
*Cratageus gracilior*	Corsolic acid	Ursane	Vasodilatory effect on rat aorta: EC_50_ 108.9 ± 6.7 μM, E_max_ 96.4 ± 4.2%	[43]
*Galphimia glauca*	Galphimidin	Nor-seco friedelane	Vasodilatory effect on rat aorta: EC_50_ 145.9 ± 6.7 μM, E_max_ 99.5 ± 5.3%	
*Jatropha neupaciflora*	3β-trans-p-coumaroyl-oxy-16-β-hydroxy-20(29)-lupene	Lupane	Vasodilatory effect on rat aorta: 63.2 ± 5.8 μM EC_50_ and 27.5 ± 1.9%	
*Sebastiania adenophora*	3-*epi*-β-amyrin	Ursane	Approximate effect on root growth at 250 μg/mL • *Lycopersicon esculentum*: −21% • *Echinocloa crus-galli*: −36% • *Amaranthus hypochondriacus*: +55%	[44]
	β-amyrinone	Ursane	Approximate effect on root growth at 250 μg/mL • *L. esculentum*: −28% • *E. crus-galli*: −28% • *A. hypochondriacus*: +39%	
	3-*epi*-lupeol	Lupane	Approximate effect on root growth at 250 μg/mL • *L. esculentum*: −32% • *E. crus-galli*: −9% • *A. hypochondriacus*: +47%	
	Lupenone	Lupane	Approximate effect on root growth at 250 μg/mL • *L. esculentum*: −36% • *E. crus-galli*: −5% • *A. hypochondriacus*: +27%	
	Taraxerol	Taraxerane	Approximate effect on root growth at 250 μg/mL • *L. esculentum*: −41% • *E. crus-galli*: −77% • *A. hypochondriacus*: +39%	
	Taraxerone	Taraxerane	Approximate effect on root growth at 250 μg/mL • *L. esculentum*: −49% • *E. crus-galli*: −44% • *A. hypochondriacus*: +23%	
*Galphimia glauca*	Glaucacetalin E	Nor-seco friedelane	Anxiolytic effect on mice at 1, 10, and 30 mg/kg dose	[45]
	Galphimidin B		Anxiolytic effect on mice at 1, 10, and 30 mg/kg dose	
	Galphimidin		Anxiolytic effect on mice at 1, 10, and 30 mg/kg dose	

* MIC: Minimum inhibitory concentration; IC_50_: 50% inhibitory concentration; ID_50_: 50% inhibitory dose; EC_50_: Half maximal effective concentration; Emax: maximum effect, H37Rv: M. tuberculosis-sensitive strain to rifampicin, streptomycin, isoniazid, and ethambutol; STR: streptomycin; RIF: rifampicin; INH: isoniazid; EMB: ethambutol; MMDO: M. tuberculosis strain resistant to isoniazid and ethambutol; MTY 147: M. tuberculosis resistant to isoniazid, ethambutol, rifampicin, and ethionamide; CFU: colony-forming unit; NO: nitric oxide; PC-3: human prostate tumor cell line; K562: human leukemia cell line; HCT-15: human colon tumor cell line; MCF-7: human breast tumor cell line; SKLU-1: human lung tumor cell line; MCF-7: human breast tumor cell line; U251: human glioblastoma cell line.

## Data Availability

Not applicable.

## Supplementary Materials

The following supporting information can be downloaded at: https://www.mdpi.com/article/10.3390/plants11172184/s1, Table S1: Estimated dielectric constants for solvent systems used in column chromatography (CC) for the purification of PCTs from Mexican plants.
