# Supplementary material for: State-of-the-Art and Opportunities for Bioactive Pentacyclic Triterpenes from Native Mexican Plants
Source: Plants (Basel). 2022 Aug 23;11(17):2184. doi: 10.3390/plants11172184 (PMC9459852; doi:10.3390/plants11172184)
Supplement: Supplementary file 1 [file plants-11-02184-s001.zip › plants-1847266-supplementary.pdf]

Supplementary material

This section contains an estimation of the dielectric constants presented on column chromatography systems for the purification of PCTs.

Table S1. Estimated dielectric constants for solvent systems used in column chromatography (CC) for the purification of PCTs from Mexican plants.

Solvent systems (%)	Estimated Dielectric Constant *	Reference
Hexane:ethyl acetate (27.7:72.3)	4.85	[34]
Hexane:acetone (85:15)	4.76	[42]
Hexane:acetone (65:35)	8.58	[42]
Hexane:dichlorometane (30:70)	6.82	[44]
Hexane:methanol (50:50)	17.44	[29,33]
Hexane:dichlorometane:methanol (30:25:25)	11.43	[30]
Trichlorometane:methanol (80:20)	10.45	[36]
Dichlorometane:methanol (55:45)	19.76	[37]

*Estimated as weighted considering the dielectric constant for individual solvents from [51].
